# Early mortality prediction after severe trauma using ensemble machine learning: a single-center retrospective study

**DOI:** 10.3389/fpubh.2025.1716910

**Published:** 2025-12-18

**Authors:** Lijing Ling, Jin Ma, Xiaohua Xia, Hua Yuan, Shifang Liu, Yuelan Zhu

**Affiliations:** 1 Department of Emergency Medicine, Affiliated Kunshan Hospital of Jiangsu University, Kunshan, China; 2 Department of Nursing, Affiliated Kunshan Hospital of Jiangsu University, Kunshan, China

**Keywords:** emergency, mortality, trauma, machine learning, retrospective study

## Abstract

Early identification of trauma patients at risk of in-hospital death is essential for guiding time-critical resuscitation and operative strategies. We developed and evaluated a multi-model machine learning framework that integrates vital signs, and routine laboratory and blood-gas metrics indices obtained within 30 min of emergency department arrival to predict in-hospital mortality. This single-center retrospective study included 408 critically injured trauma patients treated at the Emergency Department of the Affiliated Kunshan Hospital of Jiangsu University (January 2020–December 2021). We implemented multiple machine learning models [logistic regression, Random Forest, Gradient Boosting, XGBoost, LightGBM, and multilayer perceptron (MLP)], and constructed stacking and soft-voting ensembles. On the test set, single-model AUROC ranged from 0.743 to 0.927, with corresponding AUPRC spanning 0.438 to 0.904. The stacking ensemble achieved AUROC 0.9462 and AUPRC 0.8679; the voting ensemble achieved AUROC 0.9506 and AUPRC 0.8715. Permutation importance in the stacking model prioritized Injury Severity Score (ISS) (mean AUROC decrease 0.0360), base excess (BE) (0.0258), Glasgow Coma Scale (GCS) (0.0247), and pH (0.0153). In conclusion, an ensemble machine learning approach integrating early vital signs and laboratory data provides excellent discrimination and calibration for in-hospital mortality after severe trauma, with dominant contributions from ISS, GCS, and acid–base variables. These findings support the feasibility of interpretable ensemble learning for early mortality risk stratification in severe trauma.

## Introduction

Severe trauma continues to be a leading cause of death and disability globally, particularly among younger individuals, and it imposes extensive social and economic burdens through loss of productivity and prolonged recovery (1–3). In the acute phase, rapid and accurate triage is vital to prioritize transport, imaging, damage-control surgery, and massive transfusion protocols. Traditional tools remain indispensable. The Glasgow Coma Scale (GCS) quantifies neurologic impairment and is associated with survival and functional outcomes (4–6). The Injury Severity Score (ISS) provides an anatomical summary of injury burden and correlates with mortality and resource consumption (7–9). The Shock Index (SI), derived from the ratio of heart rate to systolic blood pressure, is a pragmatic marker of hemodynamic compromise and hidden shock, often outperforming single vital signs (10, 11). Beyond these scores, early laboratory indicators—especially blood lactate, pH, and base excess (BE)—reflect tissue hypoperfusion and metabolic acidosis and have been repeatedly linked to adverse outcomes in trauma and critical illness (12, 13).

The availability of rapid blood-gas analyzers and emergency laboratory testing has made it possible to capture a multi-dimensional physiological snapshot within minutes of arrival (14, 15). This context lends itself to modern machine learning (ML), which can model complex nonlinearities and interactions to deliver individualized risk predictions. In multiple settings, ML approaches have surpassed conventional scores in predicting mortality, massive transfusion, and critical care needs (16–18). Yet translation into practice is hampered by concerns over transparency and generalizability. Explainability methods such as SHAP (Shapley Additive Explanations) allow granular interpretation of model behavior, bridging the gap between performance and trust (19–21).

The present study addresses this gap by developing and explaining a comprehensive ML pipeline for early in-hospital mortality prediction among trauma patients in a Grade A tertiary hospital. We hypothesized that an ensemble of diverse learners trained on routinely gathered vital signs, blood-gas parameters, and laboratory indices would achieve excellent discrimination and calibration. We further anticipated that the most influential predictors would align with established trauma physiology—particularly injury severity, neurologic compromise, acidosis, and coagulopathy—thus enhancing interpretability and clinical acceptability.

## Methods

### Study design and setting

We conducted a single-center retrospective cohort study at the Emergency Department of the Affiliated Kunshan Hospital of Jiangsu University, Jiangsu Province, China. A total of 408 adult patients with multiple traumatic injuries who were critically ill at presentation were enrolled between January 2020 and December 2021. Most patients were transported due to traffic collisions, falls, or other high-energy mechanisms. Care pathways included damage-control surgery for severe thoracic or abdominal injury, and interventional radiology or pelvic packing for pelvic fracture–related post-peritoneal hemorrhagic shock, following local protocols. The study was approved by the institutional ethics committee (No. 2025-03-022-H00-K01).

### Data collection and variables

Demographics and admission classification were abstracted from the electronic medical record. Upon emergency department arrival, vital signs (systolic blood pressure [SBP, mmHg], diastolic blood pressure [DBP, mmHg], heart rate [HR, beats/min], respiratory rate [RR, breaths/min], and peripheral oxygen saturation [SpO₂, %]) were recorded immediately during initial assessment before any interventions. Neurologic status was assessed using the Glasgow Coma Scale (GCS, range 3-15). Hemodynamic status was quantified by the Shock Index (SI = HR/SBP, unitless). Injury severity was retrospectively calculated using the Injury Severity Score (ISS, range 0-75) after definitive imaging.

Within 30 min of ED arrival and before any major therapeutic interventions (fluid resuscitation, blood product administration, or vasopressor therapy), arterial blood was obtained via radial or femoral arterial line for blood gas analysis on an ABL90 FLEX analyzer (Radiometer, Denmark), yielding pH, lactate (mmol/L), and base excess (BE, mmol/L). Simultaneously, venous blood sampling was performed for emergency laboratory measurements, including white blood cell count (WBC, ×10^9^/L), hemoglobin (HB, g/L), platelet count (×10^9^/L), prothrombin time (PT, seconds), international normalized ratio (INR, unitless), activated partial thromboplastin time (APTT, seconds), D-dimer (μg/mL FEU), fibrinogen (FIB, g/L), glucose (mmol/L), and albumin (g/L). Our institutional trauma activation protocol mandates complete collection of all these variables for every severely injured patient, resulting in zero missing data across all 408 participants.

The 30-min data collection window was selected because: (1) it aligns with the “golden hour” concept in trauma resuscitation; (2) rapid point-of-care blood gas analyzers provide results within 2–3 min; (3) our emergency laboratory has a 15-min turnaround time for stat trauma panels; (4) it ensures measurements reflect initial pathophysiology rather than treatment effects. All predictor variables were collected before the outcome (in-hospital mortality) occurred, ensuring appropriate temporal sequence and eliminating reverse causation bias.

### Statistical analysis

The analytical pipeline included standardized preprocessing, modeling, model validation, and interpretability. The complete dataset (*n* = 408 patients) was divided into training (*n* = 306, 75%) and testing (*n* = 102, 25%) subsets using stratified random sampling to preserve outcome class proportions (84.3% survivors, 15.7% non-survivors in both sets). Patient-level independence between training and test sets was rigorously maintained with zero overlap. All data preprocessing steps were performed exclusively on the training set, with derived parameters then applied to the test set.

Model development encompassed multiple base learners: logistic regression, Random Forest, Gradient Boosting, XGBoost, LightGBM, and multilayer perceptron (MLP). Two meta-ensemble methods were constructed: (1) Stacking ensemble integrating all six base models as first-level learners with logistic regression (max_iter = 500, class_weight = “balanced”) as the meta-learner, combining predictions via “predict_proba”; (2) Soft-voting ensemble aggregating predicted probabilities from the six base models using cross-validated AUROC-derived weights. Model performance was rigorously evaluated through: (1) 5-fold cross-validation; (2) Test set evaluation (*n* = 102).

Discrimination was quantified using: area under the receiver operating characteristic curve (AUROC), area under the precision–recall curve (AUPRC), accuracy, precision, recall, and F1-score. Calibration was assessed using: calibration curves (plotting observed vs. predicted probabilities in deciles) and Brier score (mean squared error between predicted probabilities and observed outcomes, range 0–1, lower is better). Model explanations were derived from native feature importance for tree-based learners and from permutation importance applied to the stacking ensemble, complemented by SHapley Additive exPlanations (SHAP)-based visualizations. Decision curve analysis (DCA) was used to standard net benefit calculation across threshold probabilities, and assess clinical utility, comparing model-guided decisions against “treat all” and “treat none” strategies.

## Results

### Participant characteristics

A total of 408 trauma patients were included in this analysis. The cohort comprised 343 survivors (84.07%) and 65 non-survivors (15.93%). On arrival, systolic and diastolic blood pressure medians were 112.0 and 71.5 mmHg; heart rate and respiratory rate medians were 91.5 bpm and 21 breaths/min. Median SpO2 was 95.5%. The Glasgow Coma Scale (GCS) had a median of 13. The Injury Severity Score (ISS) median was 23. Within 30 min, arterial blood gas showed a median pH of 7.36, lactate 2.30 mmol/L, and base excess −4.1 mmol/L. The Shock Index (SI) median was 0.856 (Table 1).

**Table 1:** Descriptive statistics for trauma patients (*N* = 408).

Characteristics	Median (Min-Max)	Mean ± SD/*n* (%)
Age (years)	50 (18–90)	49.02 ± 16.84
Gender		
Male		272 (66.7%)
GCS	13 [3–15]	11.8 ± 4.09
Heart rate, bpm	91.5 [35–169]	94.42 ± 23.69
Respiratory rate, breaths/min	21 [0–42]	21.88 ± 6.28
SBP, mmHg	112 [35–215]	112.24 ± 31.47
DBP, mmHg	71.5 [20–128]	69.06 ± 20.9
SpO2, %	95.5 [50–100]	92.31 ± 8.49
pH	7.36 [6.82–7.57]	7.33 ± 0.12
Lactate, mmol/l	2.3 [0.4–16]	3.48 ± 3.3
BE, mmol/l	−4.1 [−25.6 to 8.7]	−5.22 ± 5.6
Hemoglobin, g/l	130.5 [29–187]	125.92 ± 27.38
WBC, x10^9^/l	13.24 [3.65–41.1]	14.92 ± 6.95
Lymphocyte, x10^9^/l	2.825 [0.14–9.43]	3.14 ± 1.91
Platelet, x10^9^/l	208.5 [20–429]	215.44 ± 82.76
Glucose, mmol/l	8.85 [1.1–38.4]	9.92 ± 4.44
Albumin, g/L	39.25 [10–52.8]	37.68 ± 7.21
Prothrombin time, sec	11.8 [0–25.3]	12.24 ± 2.86
APTT, sec	26.9 [0–225.9]	30.52 ± 16.51
INR	1.01 [0–2.23]	1.06 ± 0.24
D-Dimer	19.9 [0–291.56]	33.08 ± 37.0
FIB	1.92 [0–6.6]	2.0 ± 0.88
ISS	23 [11–72]	26.3 ± 13.83
SI	0.86 [0.31–2.88]	0.93 ± 0.42
Mortality rate		57 (16.96%)

GCS, Glasgow Coma Scale; INR, International Normalized Ratio; ISS, Injury Severity Score; SBP, Systolic Blood Pressure; SI, Shock Index; SpO2, Peripheral Oxygen Saturation; WBC, White Blood Cell Count.

Compared to survivors, non-survivors had significantly more severe injuries as evidenced by higher ISS (47.11 ± 13.97 vs. 21.54 ± 8.82, median 46 vs. 18, *p* < 0.001) and more profound neurologic impairment (GCS: 5.52 ± 3.39 vs. 13.15 ± 2.83, median 4 vs. 15, *p* < 0.001) (Supplementary Table S1). Non-survivors also exhibited worse hemodynamic compromise with lower blood pressure (SBP: 90.52 ± 35.69 vs. 118.48 ± 28.63 mmHg, *p* < 0.001; DBP: 53.02 ± 24.10 vs. 73.38 ± 18.59 mmHg, *p* < 0.001), higher shock index (SI: 1.26 ± 0.57 vs. 0.85 ± 0.35, *p* < 0.001), and impaired oxygenation (SpO₂: 83.48 ± 11.08% vs. 94.45 ± 6.23%, *p* < 0.001).

### Model performance

Six individual models were trained and evaluated, including logistic regression, Random Forest, Gradient Boosting, XGBoost, LightGBM, and MLP. On the test set, Random Forest demonstrated AUROC = 0.927 and AUPRC = 0.904, LightGBM reached AUROC = 0.910 and AUPRC = 0.863, XGBoost attained AUROC = 0.872 and AUPRC = 0.842, Gradient Boosting showed AUROC = 0.834 and AUPRC = 0.821, Logistic Regression achieved AUROC = 0.917 and AUPRC = 0.792, and MLP yielded AUROC = 0.743 and AUPRC = 0.438 (Table 2). Cross-validation results (Supplementary Table S2) showed consistent performance hierarchy, with mean AUROC values of 0.979 for Random Forest, 0.948 for XGBoost, 0.964 for LightGBM, 0.961 for Logistic Regression, 0.934 for Gradient Boosting, and 0.862 for MLP.

**Table 2:** Single-model and meta-ensemble performance on test sets.

Model	AUROC	AUPRC	Accuracy	Precision	Recall	F1-score	Brier score
Random forest	0.9273	0.9040	0.9510	1.0000	0.6875	0.8148	0.0440
Logistic regression	0.9172	0.7923	0.9020	0.6875	0.6875	0.6875	0.0704
LightGBM	0.9099	0.8635	0.9412	0.9167	0.6875	0.7857	0.0509
XGBoost	0.8721	0.8416	0.9314	0.8462	0.6875	0.7586	0.0518
Gradient boosting	0.8343	0.8215	0.9510	0.9231	0.7500	0.8276	0.0490
MLP	0.7427	0.4379	0.8529	1.0000	0.0625	0.1176	0.1409
Stacking ensemble	0.9462	0.8679	0.9412	0.8571	0.7500	0.8000	0.0496
Voting ensemble	0.9506	0.8715	0.9510	0.9231	0.7500	0.8276	0.0482

### Ensemble performance

**Figure 1:**
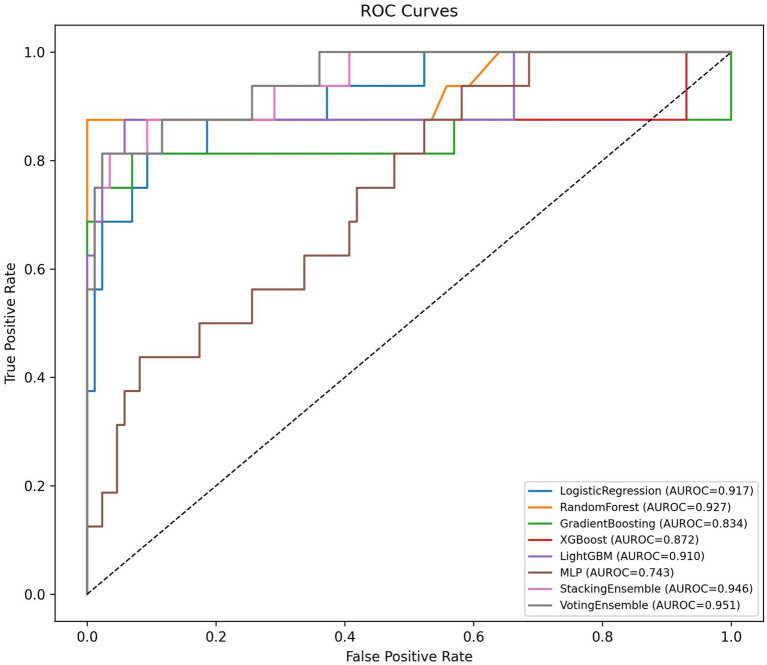
ROC curves of different individual machine learning models.

The stacking ensemble achieved AUROC = 0.946, AUPRC = 0.868, accuracy = 0.941, precision = 0.857, recall = 0.750, F1 = 0.800, and Brier score = 0.050 (Table 2; Figure 1). The soft-voting ensemble attained AUROC = 0.951, AUPRC = 0.872, accuracy = 0.951, precision = 0.923, recall = 0.750, F1 = 0.828, and Brier score = 0.048 (Table 2; Figure 1). Both meta-ensembles maintained near-peak discrimination while providing well-calibrated probability estimates, as evidenced by low Brier scores. Calibration curves demonstrated agreement between predicted probabilities and observed frequencies across all deciles (Supplementary Figure S1).

### Feature importance and model interpretability

**Figure 2:**
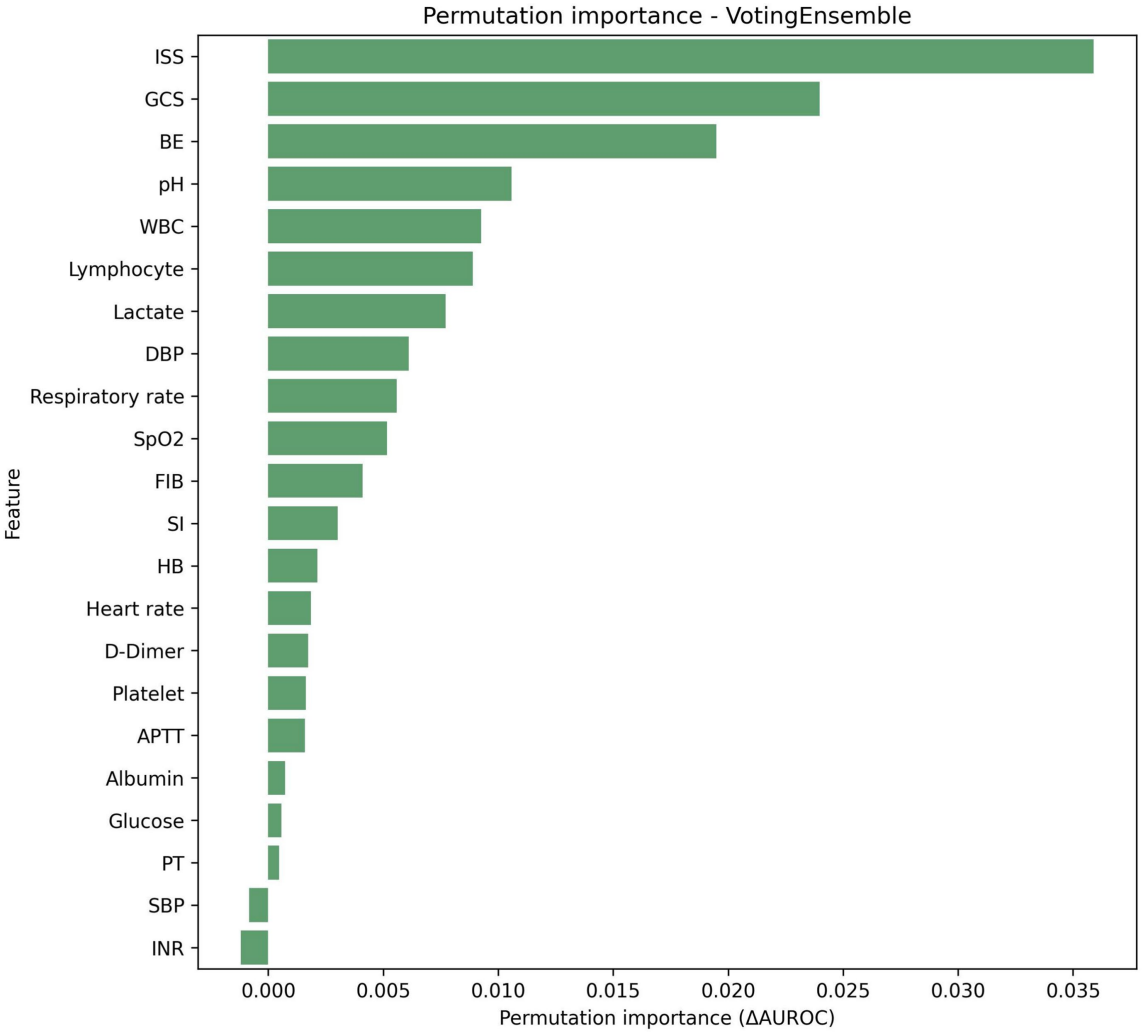
Permutation importance for the best model (voting ensemble).

**Figure 3:**
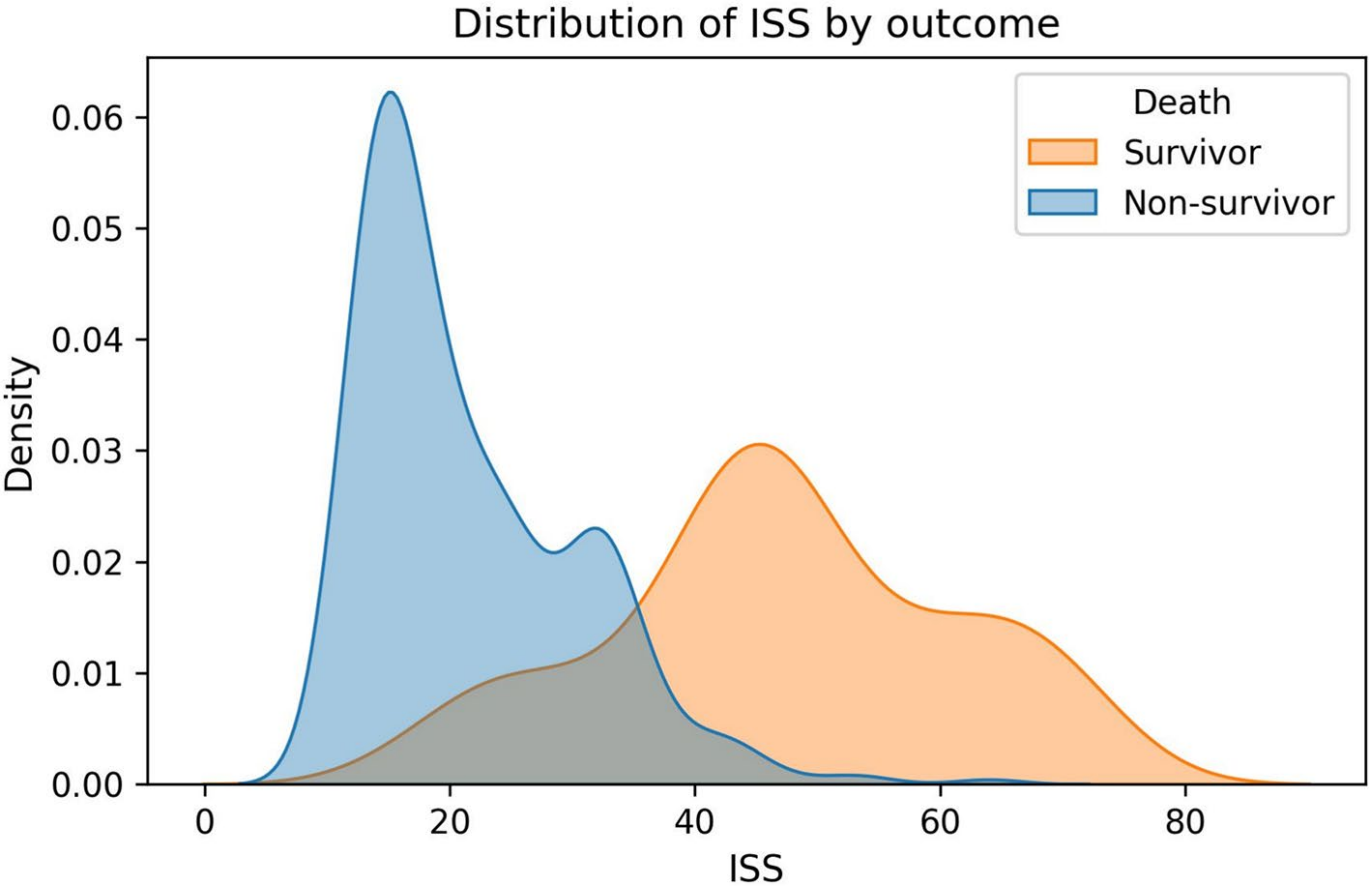
The distribution difference of ISS between deceased patients and surviving patients.

**Figure 4:**
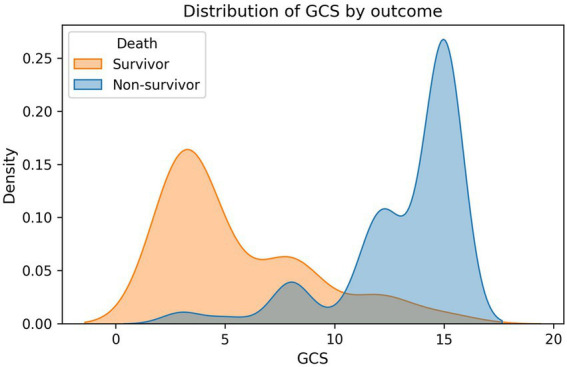
The distribution difference of GCS between deceased patients and surviving patients.

Permutation importance analysis on the stacking ensemble confirmed ISS (0.0360 ± 0.0128), BE (0.0258 ± 0.0116), GCS (0.0247 ± 0.0171), pH (0.0153 ± 0.0072), and lactate (0.0136 ± 0.0056) as the leading contributors (Table 3). Permutation importance for the voting ensemble similarly highlighted ISS (0.0359 ± 0.0132), GCS (0.0240 ± 0.0133), BE (0.0195 ± 0.0089), and pH (0.0106 ± 0.0054) as dominant predictors (Table 4; Figures 2–4). Physiological variables (SpO₂, SI, blood pressure) and coagulation markers (FIB, albumin, D-dimer, PT, APTT) showed smaller but positive importance values, while some hematologic parameters (WBC, platelet, HB) exhibited near-zero or slightly negative permutation importance.

**Table 3:** Threshold analysis for the voting ensemble.

Threshold	Accuracy	Precision	Recall	F1 score
0.1	0.843	0.500	0.875	0.636
0.2	0.941	0.813	0.813	0.813
0.3	0.941	0.857	0.750	0.800
0.4	0.951	0.923	0.750	0.828
0.5	0.951	0.923	0.750	0.828
0.6	0.941	0.917	0.688	0.786
0.7	0.922	0.900	0.563	0.692
0.8	0.922	1.000	0.500	0.667
0.9	0.853	1.000	0.063	0.118

**Table 4:** Permutation importance for the best model (voting ensemble).

Feature	Importance mean	Importance std
ISS	0.0359	0.0132
GCS	0.0240	0.0133
BE	0.0195	0.0089
pH	0.0106	0.0054
WBC	0.0093	0.0049
Lymphocyte	0.0089	0.0049
Lactate	0.0077	0.0027
DBP	0.0061	0.0060
Respiratory rate	0.0056	0.0065
SpO2	0.0052	0.0054
FIB	0.0041	0.0042
SI	0.0030	0.0025
HB	0.0021	0.0021
Heart rate	0.0019	0.0041
D-Dimer	0.0017	0.0015
Platelet	0.0016	0.0014
APTT	0.0016	0.0020
Albumin	0.0007	0.0021
Glucose	0.0006	0.0020
PT	0.0005	0.0024
SBP	−0.0008	0.0025
INR	−0.0012	0.0047

**Figure 5:**
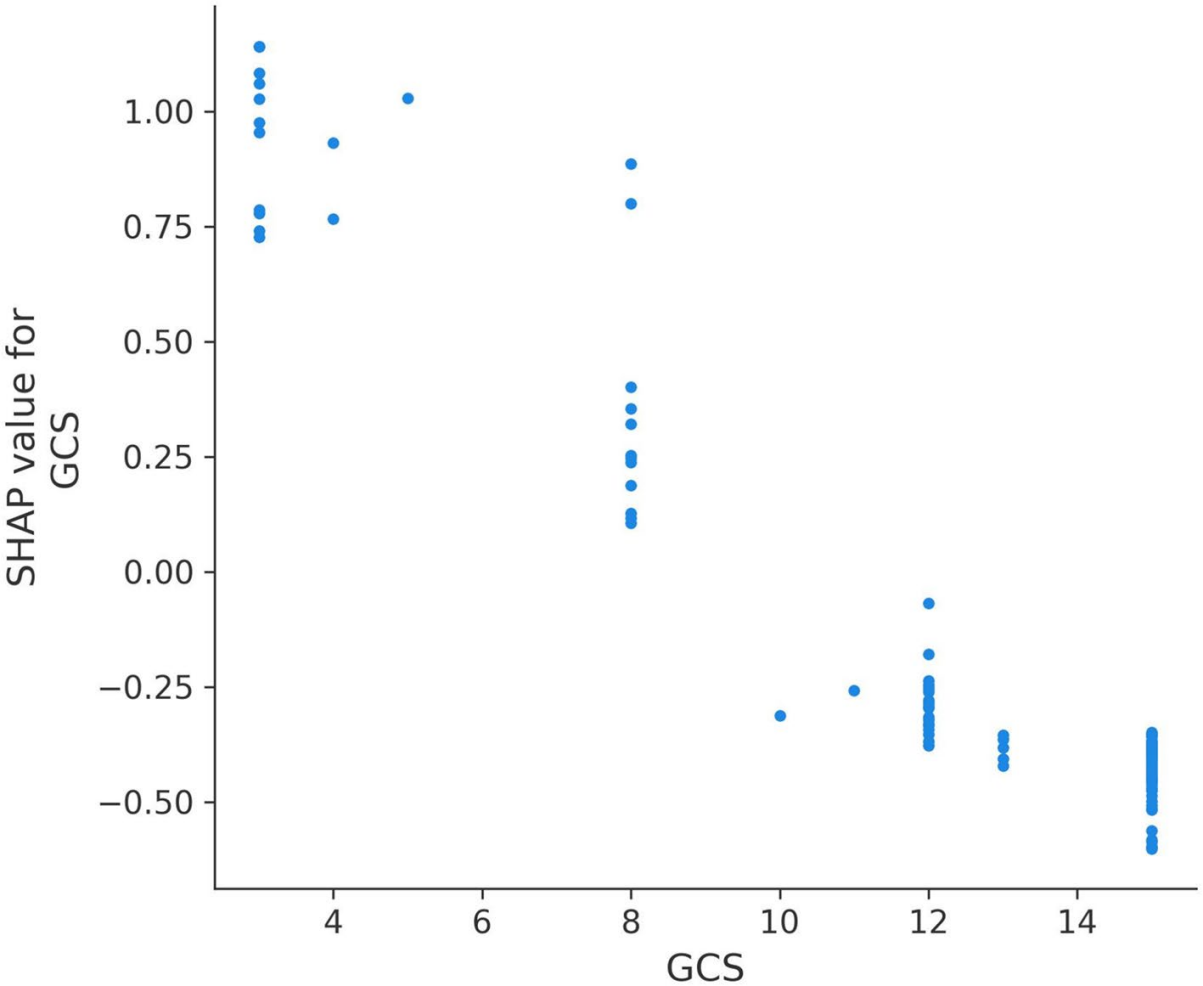
SHAP summary plots for GCS.

**Figure 6:**
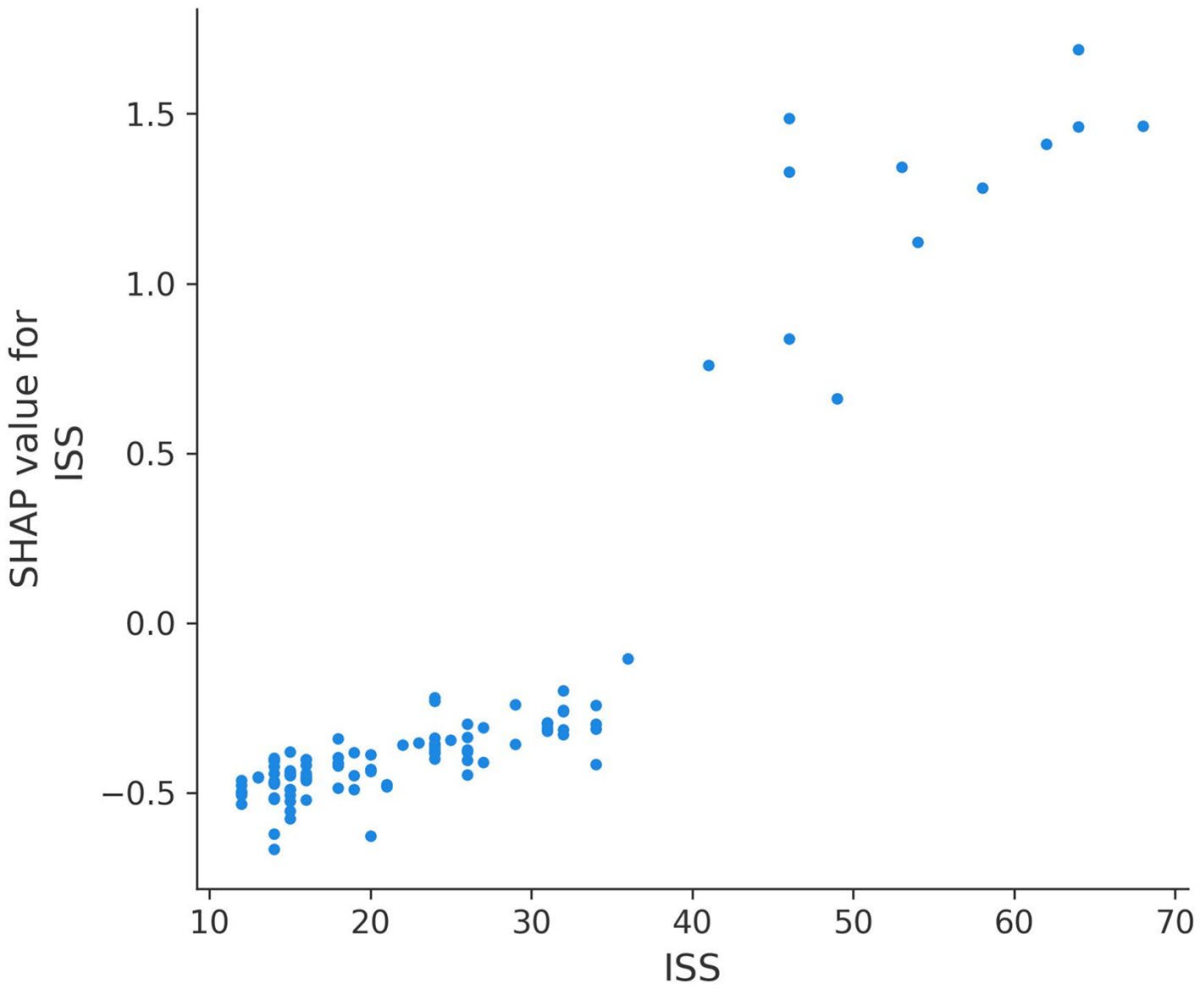
SHAP summary plots for ISS.

**Figure 7:**
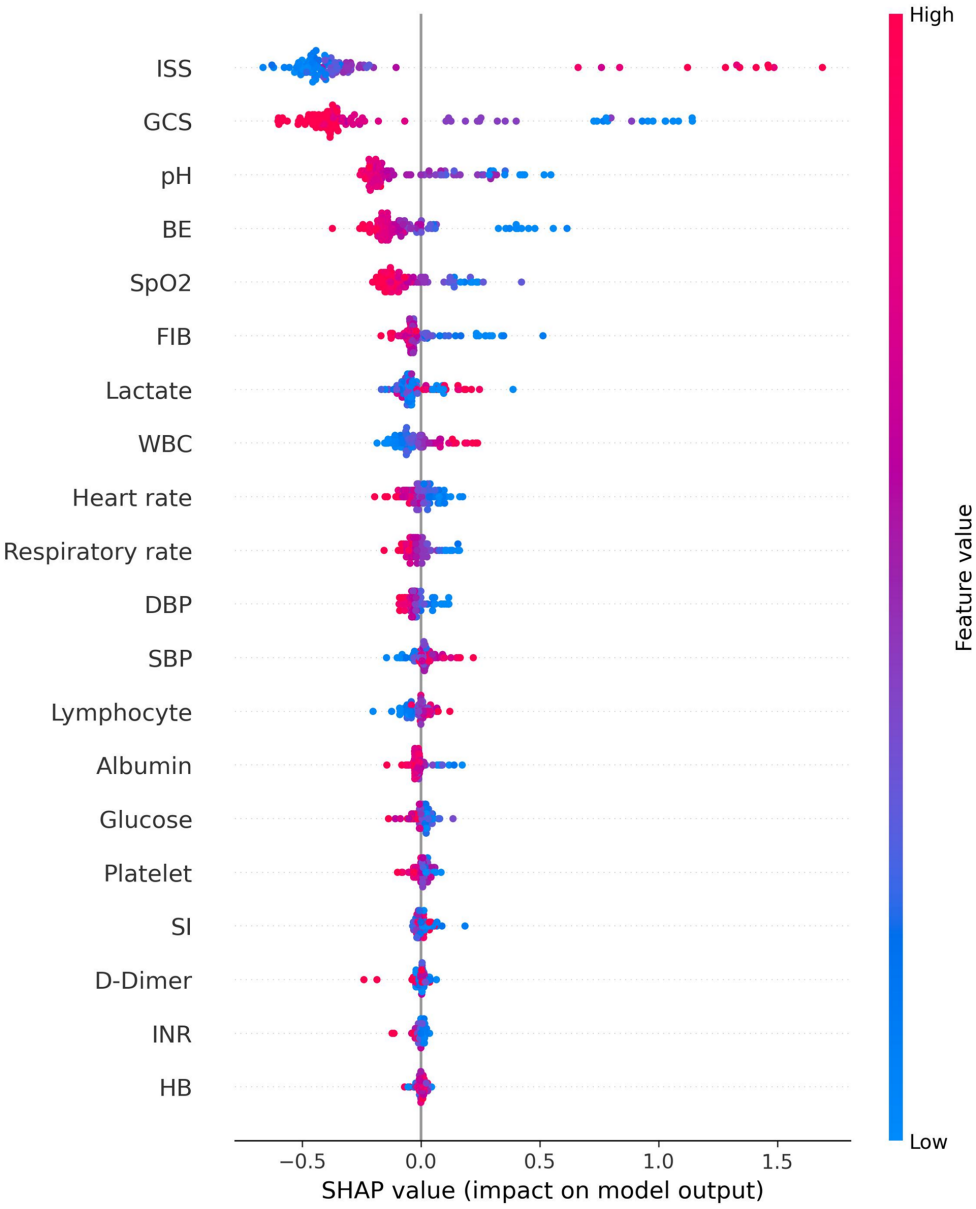
SHAP bar plots for the best model (voting ensemble).

SHAP analysis provided granular insights into how each variable influenced individual predictions. SHAP summary plots demonstrated that low GCS values (especially <8) consistently increased mortality risk across patients. High ISS (>40) sharply elevated risk (Figures 5, 6). SHAP bar plots (global feature importance) confirmed the ranking: ISS, GCS, pH, BE, and Lactate dominated across all models (Figure 7). These interpretability analyses demonstrate that the models’ predictions are driven by clinically established mortality risk factors in trauma: anatomic injury severity (ISS), neurologic impairment (GCS), metabolic acidosis (pH, BE, lactate), coagulopathy (FIB, PT, APTT, D-dimer), and hemodynamic compromise (SI, blood pressure, SpO₂).

### Decision curve analysis and clinical utility

DCA demonstrated positive net benefit for probability thresholds between 0.15 and 0.70 (Supplementary Figure S2), aligning with threshold-specific operating characteristics. At a 0.30 risk threshold, the stacking ensemble provided accuracy = 0.941, precision = 0.813, recall = 0.813, and F1 = 0.813 (Table 3). At thresholds of 0.50 and 0.70, the stacking ensemble maintained precision = 0.857 with recall = 0.750 and precision = 0.923 with recall = 0.750, respectively, supporting adaptable triage decisions.

## Discussion

This study demonstrates that ensemble machine learning models integrating early vital signs, blood gas parameters, and routine laboratory data can accurately predict in-hospital mortality among severely injured trauma patients, achieving AUROC values of 0.7427–0.9273 for single models and 0.9462–0.9506 for meta-ensembles. Comprehensive interpretability analyses demonstrated that predictions are driven by clinically established risk factors—injury severity (ISS), neurologic impairment (GCS), and metabolic acidosis (pH, BE, lactate)—enhancing clinical trustworthiness (22–24).

Our results are congruent with recent literature applying ML to trauma outcomes. Prior studies have shown that machine learning methods can outperform conventional scores and regression-based approaches for predicting mortality, leveraging their capacity to capture nonlinear and interaction effects (25–27). Tree-based ensembles and boosting frameworks, in particular, tend to provide robust performance while preserving a degree of interpretability through feature importance and SHAP-based visualizations (28, 29). In this study, even without extensive hyperparameter optimization, multiple learners achieved excellent discrimination, and ensemble methods further stabilized performance and probability estimates. The threshold analyses illustrate how quantitative risk outputs can be transformed into actionable triage rules, tailored to institutional risk tolerances and resource constraints.

Several aspects of the methodological approach warrant emphasis. The focus on variables obtainable within 30 min of ED arrival ensures temporal relevance for decision-making about operative control and resuscitation strategies. Furthermore, permutation importance on the stacked model offers a model-agnostic confirmation of feature contributions, mitigating biases from any single algorithm’s importance measure. However, this study has limitations. As a single-center retrospective analysis, it is subject to local practice patterns, case mix, and data completeness.

The clinical implications are significant. Early, accurate, and interpretable mortality risk estimates can support activation of high-intensity protocols, prioritization for operating room or interventional radiology, and allocation of ICU beds. The ability to tune risk thresholds provides a mechanism to adapt to surge conditions or limited resources without reconfiguring the model. Because the features are already part of routine ED workflows, implementation barriers are relatively low. Embedding the model into a clinical decision support dashboard that surfaces both the risk score and its leading contributors would allow clinicians to cross-check outputs against bedside assessment and to identify potential reversible drivers—such as acidosis or hypoxemia—for targeted resuscitation.

This study has several limitations. First, the single-center retrospective design restricts generalizability to other trauma populations, healthcare systems, and geographic regions. While bootstrap confidence intervals and cross-validation demonstrate internal validity and model stability, external validation in independent cohorts is essential before clinical implementation. Second, the 30-min data collection window, while pragmatic and clinically relevant, may not capture evolving physiology during resuscitation. Some patients may deteriorate or improve substantially within the first hours, information not incorporated in our static baseline model. Third, we did not assess model fairness or equity across patient subgroups defined by age, sex, race/ethnicity, socioeconomic status, or injury mechanism. Machine learning models can perpetuate or amplify healthcare disparities if trained on biased data or if predictive features correlate with protected characteristics.

## Conclusion

In a cohort of severely injured trauma patients in China, ensemble machine learning models trained on early vital signs, blood-gas parameters, and laboratory indices achieved excellent discrimination and calibration for in-hospital mortality. The dominant predictive contributions of ISS, GCS, and markers of hypoperfusion and acid–base imbalance are consistent with established trauma physiology, enhancing the validity and interpretability of the models.

## Data Availability

The original contributions presented in the study are included in the article/Supplementary material, further inquiries can be directed to the corresponding author.
