# Supplementary material for: Early mortality prediction after severe trauma using ensemble machine learning: a single-center retrospective study
Source: Front Public Health. 2025 Dec 18;13:1716910. doi: 10.3389/fpubh.2025.1716910 (PMC12756457; doi:10.3389/fpubh.2025.1716910)

**Supplementary Tables 1. The differences in baseline characteristics between participants in the survival group and the death group.**

| **Variable** | **Survivors (Mean±SD)** | **Non survivors (Mean±SD)** | **P value** | **Significant** |
| --- | --- | --- | --- | --- |
| GCS | 13.15 ± 2.83 | 5.52 ± 3.39 | 9.90E-32 | Yes |
| Heart rate | 93.64 ± 21.86 | 99.38 ± 31.42 | 0.046327 | Yes |
| Respiratory rate | 22.50 ± 5.07 | 17.72 ± 9.16 | 1.31E-05 | Yes |
| SBP | 118.48 ± 28.63 | 90.52 ± 35.69 | 2.59E-09 | Yes |
| DBP | 73.38 ± 18.59 | 53.02 ± 24.10 | 1.87E-10 | Yes |
| SpO2 | 94.45 ± 6.23 | 83.48 ± 11.08 | 7.48E-15 | Yes |
| pH | 7.36 ± 0.07 | 7.15 ± 0.16 | 8.83E-26 | Yes |
| Lactate | 2.41 ± 1.67 | 8.33 ± 4.65 | 1.64E-23 | Yes |
| BE | -3.43 ± 3.67 | -13.27 ± 6.27 | 8.79E-26 | Yes |
| HB | 130.37 ± 24.24 | 107.82 ± 36.92 | 7.88E-06 | Yes |
| WBC | 14.99 ± 6.74 | 14.99 ± 7.87 | 0.701603 | No |
| Lymphocyte | 2.99 ± 1.84 | 3.79 ± 2.12 | 0.006689 | Yes |
| Platelet | 224.78 ± 78.13 | 177.71 ± 93.06 | 0.000205 | Yes |
| Glucose | 9.55 ± 3.86 | 11.56 ± 6.45 | 0.002563 | Yes |
| Albumin | 39.22 ± 5.61 | 31.21 ± 10.76 | 3.63E-09 | Yes |
| PT | 11.97 ± 1.85 | 13.71 ± 5.72 | 1.05E-08 | Yes |
| APTT | 27.92 ± 11.98 | 41.75 ± 24.94 | 9.65E-10 | Yes |
| INR | 1.03 ± 0.15 | 1.19 ± 0.50 | 2.82E-08 | Yes |
| D-Dimer | 28.38 ± 28.27 | 58.32 ± 55.69 | 2.75E-07 | Yes |
| FIB | 2.18 ± 0.82 | 1.25 ± 0.68 | 1.68E-16 | Yes |
| ISS | 21.54 ± 8.82 | 47.11 ± 13.97 | 6.86E-29 | Yes |
| SI | 0.85 ± 0.35 | 1.26 ± 0.57 | 1.95E-08 | Yes |

**Supplementary Table 2. Single-model performance using cross-validation.**

| **Model** | **AUROC** | **AUPRC** | **Accuracy** | **Precision** | **Recall** | **F1-score** | **Brier score** |
| --- | --- | --- | --- | --- | --- | --- | --- |
| Random Forest | 0.9789 | 0.9641 | 0.9647 | 0.9875 | 0.7875 | 0.8699 | 0.0310 |
| Logistic Regression | 0.9610 | 0.8801 | 0.9157 | 0.6832 | 0.8750 | 0.7635 | 0.0629 |
| LightGBM | 0.9638 | 0.9318 | 0.9588 | 0.9408 | 0.7875 | 0.8539 | 0.0335 |
| XGBoost | 0.9484 | 0.9172 | 0.9529 | 0.9016 | 0.7875 | 0.8373 | 0.0368 |
| Gradient Boosting | 0.9344 | 0.9156 | 0.9608 | 0.9339 | 0.8125 | 0.8606 | 0.0357 |
| MLP | 0.8616 | 0.6545 | 0.8882 | 0.8964 | 0.3375 | 0.4663 | 0.1188 |

**Supplementary Figure 1. Calibration curve for all models.**


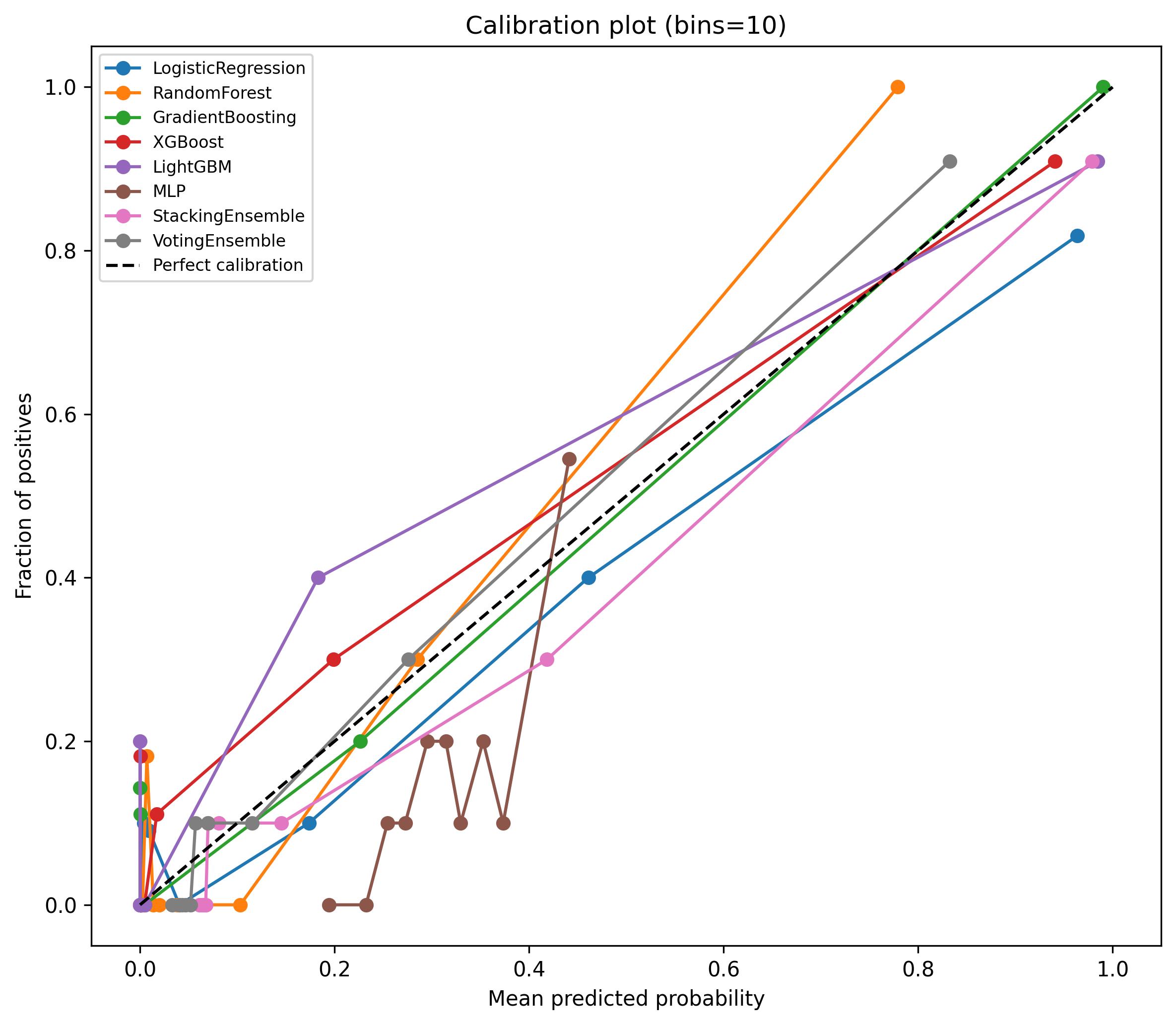


**Supplementary Figure 2. Decision curve analysis showing net benefit across threshold probabilities.**


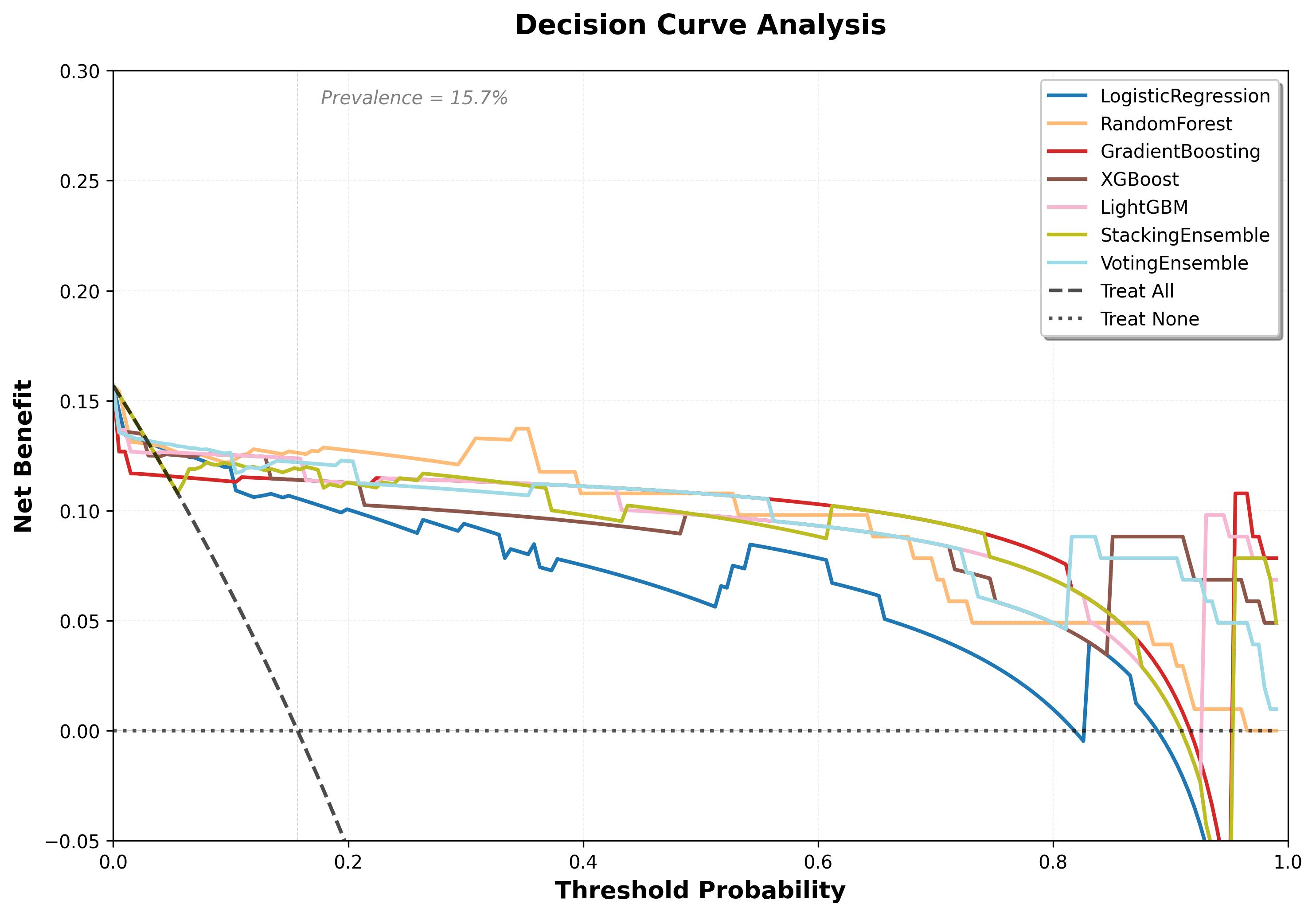

Supplement: Supplementary file 1 [file Supplementary_file_1.docx]
